# A new species of *Megachile* Latreille subgenus *Megachiloides* (Hymenoptera, Megachilidae)

**DOI:** 10.3897/zookeys.283.4674

**Published:** 2013-04-03

**Authors:** Cory S. Sheffield

**Affiliations:** 1 Royal Saskatchewan Museum, 2340 Albert Street, Regina, Saskatchewan, Canada S4P 2V7

**Keywords:** Apoidea, Anthophila, Megachilinae, Onagraceae, Texas, *Megachile chomskyi*

## Abstract

A new species of leafcutter bee, *Megachile (Megachiloides) chomskyi*, is described from Texas, United States. *Megachile chomskyi* is one of the four known species of the *oenotherae* species group of *Megachiloides*, all members sharing the long tongue, and is most similar to *Megachile (Megachiloides) amica* Cresson. Like other members of the *oenotherae* species group, this species probably shows oligolecty with Onagraceae (Evening-Primrose Family). A diagnosis, full description of both sexes and a key to the species of the *oenotherae* species group are provided.

## Introduction

**Figure 1.**
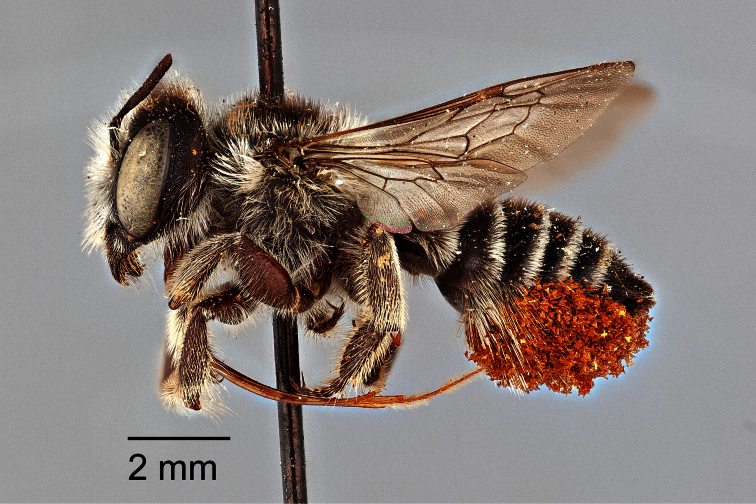
Lateral habitus of female *Megachile oenotherae* (Mitchell) (paratype); type species of *Megachiloides* Mitchell.

**Figure 2.**
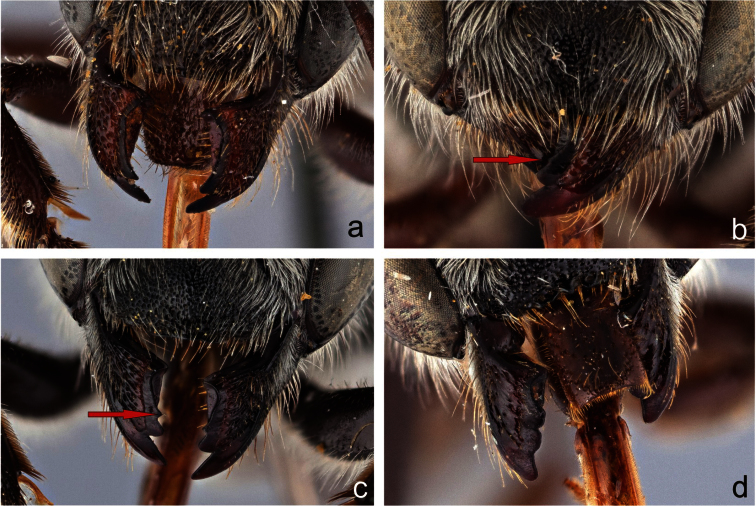
Mandibles of female *Megachile* subgenus *Megachiloides* s. l.; **a** *Megachile pascoensis* Mitchell (type species of *Derotropis* Mitchell **b** *Megachile oenotherae* (Mitchell), type species of *Megachiloides* Mitchell **c** *Megachile (Megachiloides) umatillensis* (Mitchell) **d** *Megachile integra* Mitchell, type species of the *Xeromegachile* Mitchell. Red arrows show the position of the “third” tooth in *Megachiloides* s. str.

**Figure 3.**
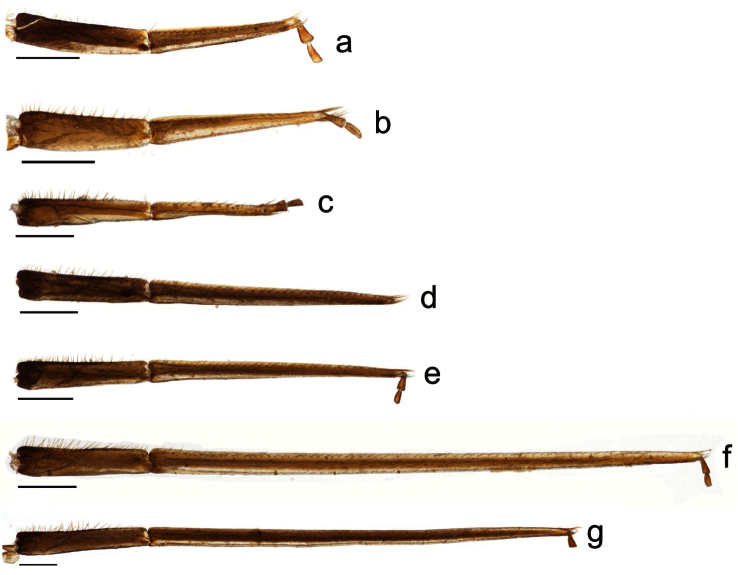
Labial palpi of *Megachile* subgenera *Megachile* (**a**) and *Megachiloides* s. l. (**b–g**), size of images adjusted to have basal palpomere of standardized length; **a** *Megachile centuncularis* (Linnaeus) (type species of *Megachile* Latreille) **b** *Megachile pascoensis* Mitchell (type species of *Derotropis* Mitchell **c** *Megachile integra* Mitchell, type species of the *Xeromegachile* Mitchell **d** *Megachile amica* Cresson **e** *Megachile chomskyi*, new species **f** *Megachile oenotherae* (Mitchell), type species of *Megachiloides* Mitchell **g** *Megachile umatillensis* (Mitchell). Scale bar = 0.5 mm.

The subgenus *Megachiloides* Mitchell is the largest in North American *Megachile* Latreille, with just under 60 described species (Michener 2007; with recent synonymies in Sheffield et al. 2011) ranging from southern Canada (Saskatchewan to British Columbia) to northern Mexico. Originally, Mitchell (1924) proposed the genus *Megachiloides* to include one species, *Megachile oenotherae* Mitchell, which was morphologically distinct in possessing an extremely elongate tongue (i.e. glossum and labial palpus) (Figures 1 and 3f), and by the characteristic 3-dentate mandible of the female, with a small additional tooth that is scarcely distinguishable from the second within the elongate interspace (Figures 2b); Michener (2000, 2007) considers this a 4-dentate condition. Subsequently, the similar (Figure 2c), albeit larger species *Megachile umatillensis* Mitchell, was added to *Megachiloides* (Mitchell 1927). Mitchell (1934) later reduced *Megachiloides* to a subgenus of *Megachile*, and added *Megachile amica* Cresson, a species which also has a relatively elongate tongue (Figures 3d) and the characteristic mandibular structure, and five additional species known at the time only from the female and possessing 3-dentate mandibles without the small tooth in the second interspace (Figure 2a), but with tongues more typical to *Megachile* (Figure 3b). Mitchell (1934) also proposed the subgenus *Xeromegachile* to include similar species with females with 4-dentate mandibles (Figure 2d) and short tongues (Figure 3c). Two years later, Mitchell (1936) proposed the subgenus *Derotropis* to separate the species with females with 3-dentate mandibles (Figure 2a) and short tongues (Figure 3b), and added several additional species, though only a few with males described and/or associated with females.

Subsequently, Mitchell (1980) reinstated *Megachiloides* to genus level; along with the subgenera *Megachiloides*, *Derotropis*, and *Xeromegachile*, he added *Argyropile* Mitchell and *Phaenosarus* Mitchell. This larger grouping thus contained all North American *Megachile* with the carina of T6 of the male being entire, excluding, at that time, members of *Argyropile* (see Mitchell 1980), though males of a few species also share this character (see Gonzalez and Griswold 2007). This broader classification thus also included females possessing 3-, 4-, and 5-dentate mandibles (Mitchell 1980). Michener (2000), not accepting Mitchell’s broad partitioning of *Megachile* (i.e. Mitchell 1980), reinstated *Megachiloides* to subgenus level and made *Derotropis* and *Xeromegachile* junior synonyms; *Argyropile* recognized again as a subgenus, and *Phaenosarus* newly synonymized under subgenus *Xanthosarus* Robertson. Thus, the subgenus *Megachiloides* s. l. contains females which are slightly less variable in mandible shape, *Megachiloides* s. str. (i.e. the *oenotherae* species group) considered an intermediate between the 3-dentate *Derotropis* (Figure 2a) and the 4-dentate *Xeromegachile* (Figure 2d), with all males with the carina of T6 entire (Michener 2000).

The objective here is to describe a new species of *Megachile* from Texas, United States, and provide a diagnosis of this species and a key to distinguish it from other members of the *oenotherae* species group in North America. This work ultimately forms a contribution to an ongoing revision and phylogeny of *Megachiloides* s. l.

## Methods

As part of previous work on *Megachile* in North America (Sheffield and Westby 2007, Sheffield et al. 2011), an ongoing revision and phylogeny of the subgenus *Megachiloides* s. l. (*sensu* Michener 2000), and a larger campaign to collect DNA barcodes from all bees (Bee-BOL; Packer et al. 2009), representatives of many species of *Megachile* were collected throughout North America and/or borrowed from other institutions. These include the Canadian Nation Collection of Insects, Arachnids and Nematodes (Ottawa, ON), the Packer Collection at York University (Toronto, ON), the Royal Saskatchewan Museum (Regina, SK), University of Alberta (Edmonton, AB) the Royal Alberta Museum (Edmonton, AB), Simon Fraser University (Vancouver, BC), USDA Bee Biology and Systematics Laboratory (Logan, UT), University of Kansas (Lawrence, KS), American Museum of Natural History (New York, NY), Central Texas Melittological Institute (Austin, TX), North Carolina State University Insect Museum (Raleigh, NC), Entomolgy Research Museum, University of California Riverside (Riverside, CA), and the USGS Patuxent Wildlife Research Center (Beltsville, MD).

Photomicrography was undertaken with a Canon EOS 5D Mark II digital camera with an MP-E 65 mm 1:2.8 1–5× macro lens. Measurements were made with an ocular micrometer on a Nikon SMZ1000 stereomicroscope. Head length was measured from the lower margin of the clypeus to vertex in facial view; tongue length was measured from the base of the prementum to the tip of the glossum. The following abbreviations are used in the descriptions: F, flagellomere; S, metasomal sternum; T, metasomal tergum; OD, median ocellar diameter; i=interspace; pd, puncture diameter. Morphological terminology generally follows Mitchell (1980) and Michener (2007).

## Systematics

### Genus *Megachile* Latreille. Subgenus *Megachiloides* Mitchell

*Megachiloides* Mitchell, 1924: 154. Type species: *Megachiloides oenotherae* Mitchell, 1924, by original designation.

*Megachile (Xeromegachile)* Mitchell, 1934: 302, 309. Type species: *Megachile integra* Cresson, by original designation.

*Megachile (Derotropis)* Mitchell, 1936: 156. Type species: *Megachile pascoensis* Mitchell, 1934, by original designation.

#### Megachile (Megachiloides) chomskyi

Sheffield sp. n.

urn:lsid:zoobank.org:act:3A56D34A-E6FE-453E-BC9E-29A19613F75D

http://species-id.net/wiki/Megachile_chomskyi

Specimen data at doi: 10.5886/txsd3at3

**Figure 4.**
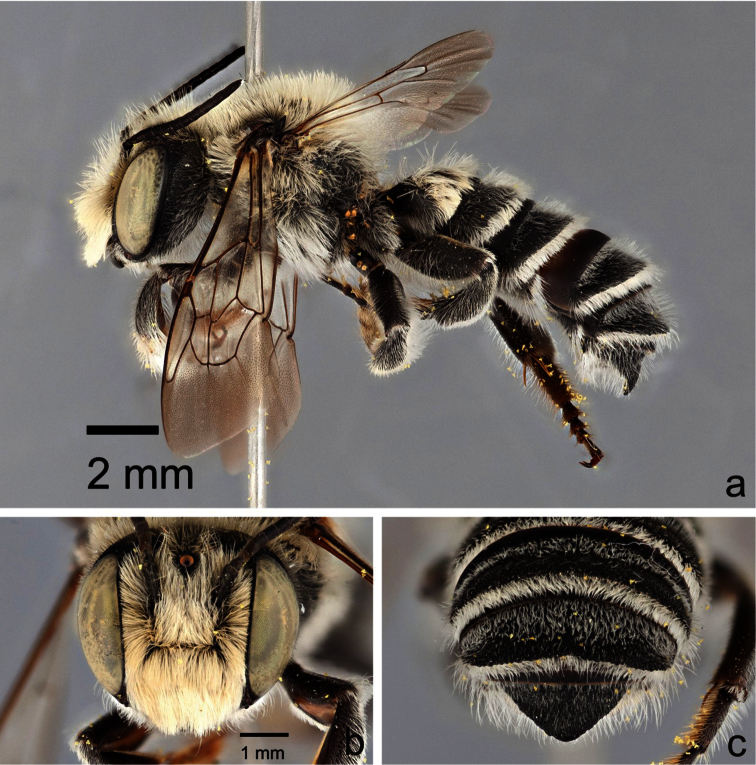
Male *Megachile (Megachiloides) chomskyi*, new species (holotype). **a** lateral habitus **b** face **c** dorsal view of terga 4–6.

**Figure 5.**
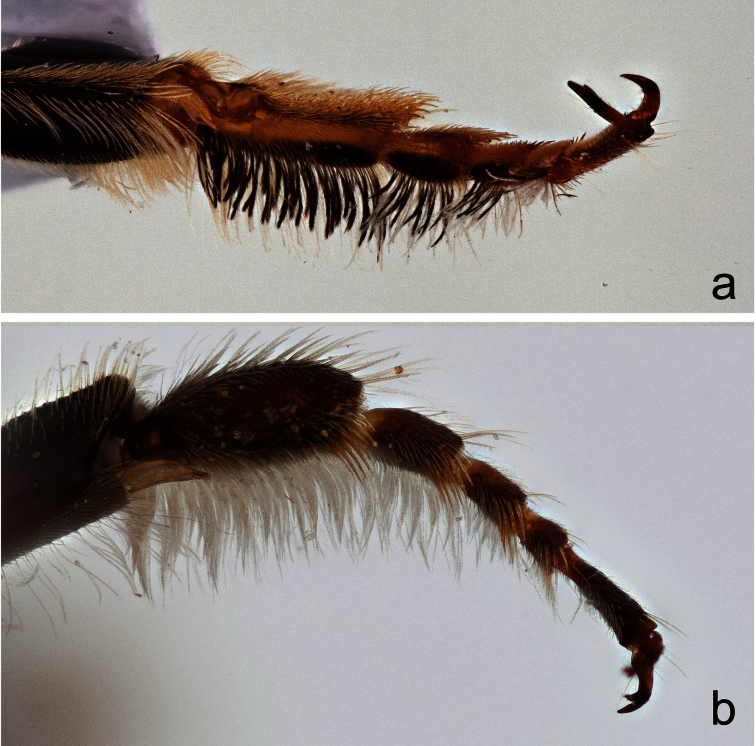
Front tarsi of male **a** *Megachile (Megachiloides) chomskyi*, new species, and **b** *Megachile (Megachiloides) oenotherae* (Mitchell).

**Figure 6.**
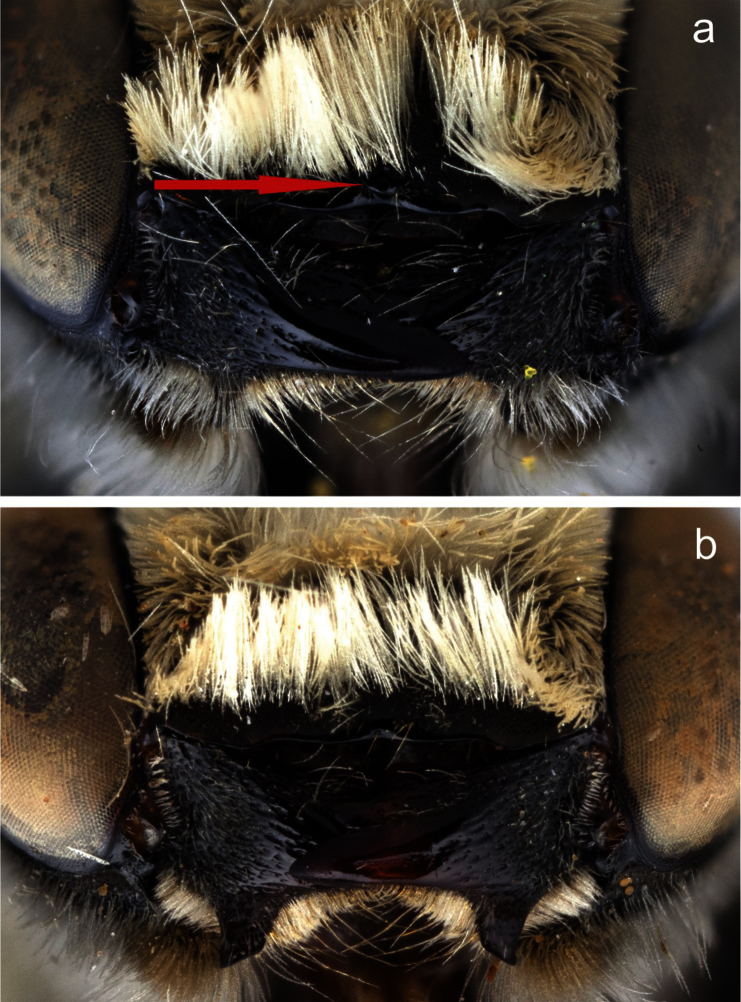
Apical edge of clypeus of male **a** *Megachile (Megachiloides) chomskyi*, new species (holotype), and **b** *Megachile (Megachiloides) amica* Cresson. Red arrow shows median tubercle.

**Figure 7.**
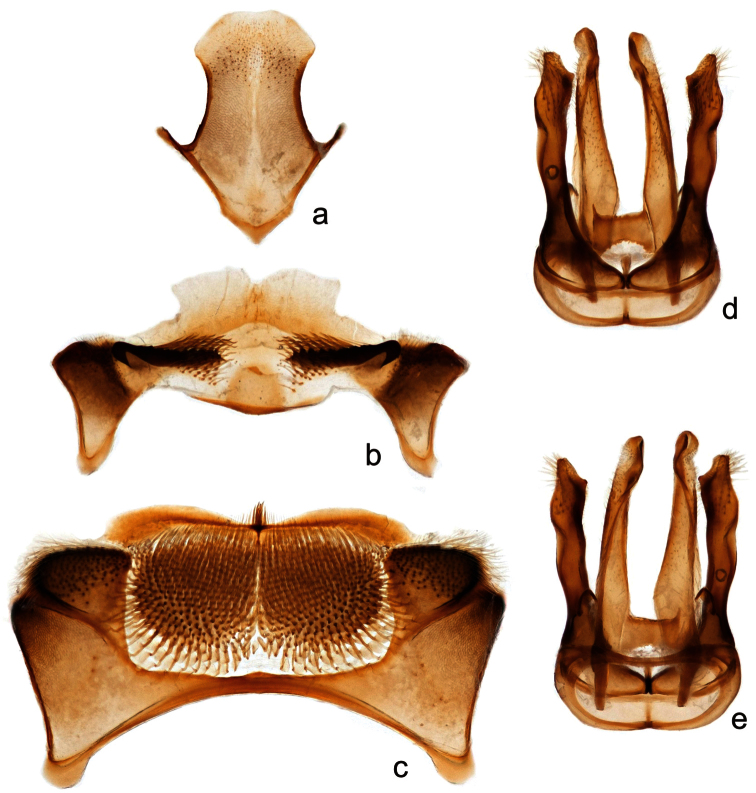
Male *Megachile (Megachiloides) chomskyi*, new species (holotype); **a** sternum 8 **b** sternum 6 **c** sternum 5, and genitalia in dorsal (**d**) and ventral (**e**) view.

**Figure 8.**
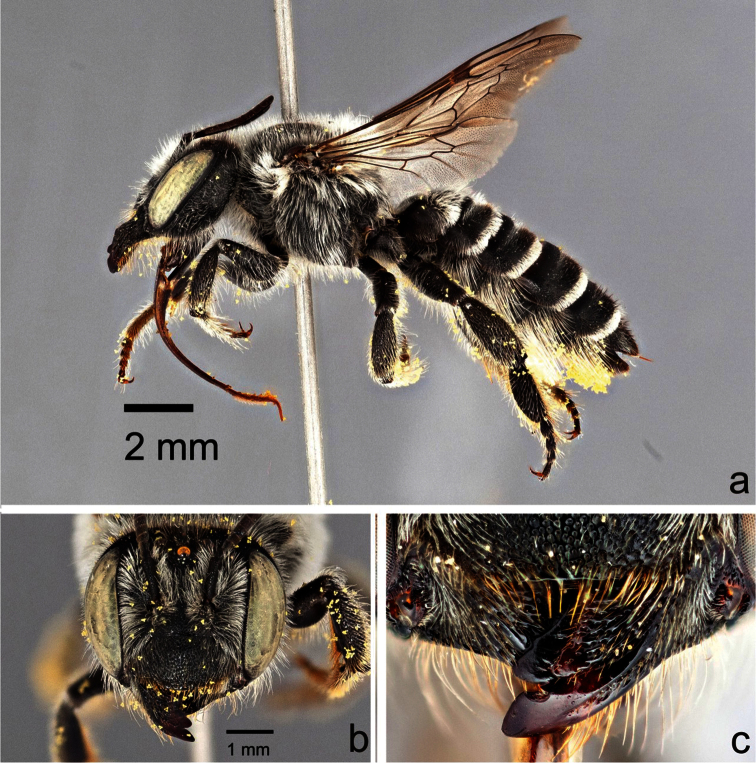
Female *Megachile (Megachiloides) chomskyi*, new species (paratype). **a** lateral habitus **b** face **c** mandible.

**Figure 9.**
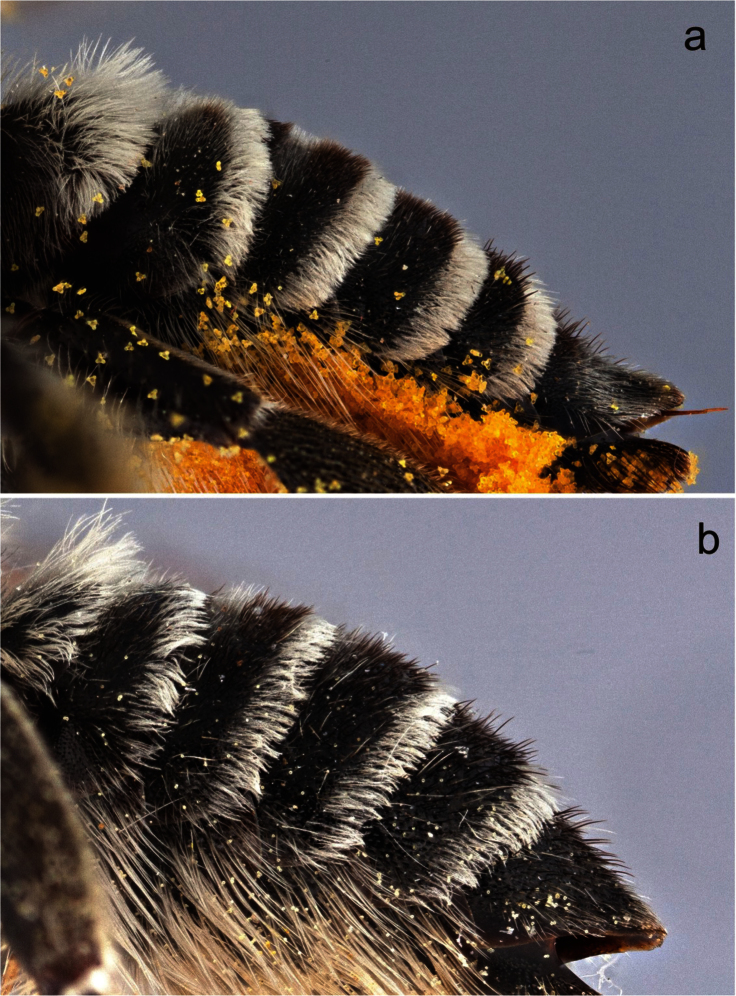
Metasomal terga of female **a** *Megachile (Megachiloides) chomskyi*, new species (paratype), and **b** *Megachile (Megachiloides) amica* Cresson.

Figures 3e , 4 , 5a , 6a , 7 , 8 , 9a

##### Holotype.

♂ (Figure 4), 29707 // TEXAS: Winkler Co., 13.2 mi. E of 18 on rd 404, 31.767°N, 102.824°W, 15-vi-2005, J. Neff & A. Hook // on flowers of *Calylophus hartweggii* // *Megachile amica* Cresson ♂, det J.L. Neff 2005 // BeeBOL, CCDB-03768 A03, BEECE003-10 [DNA barcode accession #s] // RSKM_ENT_E-0100327; deposited in the Royal Saskatchewan Museum, Regina, Saskatchewan, Canada.

**Allotype.** ♀, 29701 // TEXAS: Winkler Co., 13.2 mi. E of 18 on rd 404, 31.767°N, 102.824°W, 15-vi-2005, J. Neff & A. Hook // on flowers of *Calylophus hartweggii* // *Megachile amica* Cresson ♀, det J.L. Neff 2005 // BeeBOL, CCDB-03768 A02, BEECE002-10 [DNA barcode accession #s] // RSKM_ENT_E-0100328; deposited in the Royal Saskatchewan Museum, Regina, Saskatchewan, Canada.

##### Paratypes.

♂ TEXAS: Ward Co., Monahans Sandhills State Park, campground area, 31°38'12"N, 102°49'01"W 13 June 1998, C.R. Nelson #6733 & class // *Megachile amica* ♂, det. J. Neff 2005 // UTIC // RSKM_ENT_E-0100589; ♀, 31173 // TEXAS: Ward Co., Monahans Sandhill S. P., 31.640°N, 102.819°W, J.L. Neff, 20-v-2006 // on flowers of *Calylophus hartweggii* // *Megachile amica* Cresson ♀, det J.L. Neff 2006 // BeeBOL, CCDB-03768 A04, BEECE004-10 [DNA barcode accession #s] // RSKM_ENT_E-0100591; 2♀, 29812, 29813 // TEXAS: Ward Co., Monahans Sandhill S. P., 31.640°N, 102.819°W, 16-vi-2005, J. Neff & A. Hook // on flowers of *Calylophus hartweggii* // *Megachile amica* Cresson ♀, det J.L. Neff 2005 // RSKM_ENT_E-0100592, RSKM_ENT_E-0100593; ♀, TEXAS: Ward Co., Monahans Sandhill S. P., N31.640 W102.818, 16.VI.2005, elev 829m, A.W. Hook, J.L. Neff // *Megachile amica* Cresson ♀, det J.L. Neff 2005 // UTIC // RSKM_ENT_E-0100590; ♀, 29703 // TEXAS: Winkler Co., 13.2 mi. E of 18 on rd 404, 31.767°N, 102.824°W, 15-vi-2005, J. Neff & A. Hook // on flowers of *Calylophus hartweggii* // *Megachile amica* Cresson ♀, det J.L. Neff 2005 // BeeBOL, CCDB-03768 A01, BEECE001-10 [DNA barcode accession #s] // RSKM_ENT_E-0100579; 9♀’s, 29694-29700, 29704-29705 // TEXAS: Winkler Co., 13.2 mi. E of 18 on rd 404, 31.767°N; 102.824°W, 15-vi-2005, J. Neff & A. Hook // on flowers of *Calylophus hartweggii* // *Megachile amica* Cresson ♀, det J.L. Neff 2005 // RSKM_ENT_E-0100580-588; ♀, 21454 // TEXAS: Kleberg Co., Sarita, 3 mi. N, 27°82.94"N, 97°47.92"W, 22-iv-2001, J.L. Neff // on flowers of *Oenothera drummondii* // *Megachile amica* Cresson ♀, det J.L. Neff // RSKM_ENT_E-0100594; ♀, May, Austin, Texas // *Megachile amica* Cresson ♀, det J.L. Neff 01 // UTIC // RSKM_ENT_E-0100595. Paratypes deposited in the Royal Saskatchewan Museum, the Central Texas Melittological Institute, the University of Kansas, the USDA Bee Biology and Systematics Laboratory, and the American Museum of Natural History.

##### Diagnosis.

The male of *Megachile chomskyi* can be distinguished by the combination of the long glossum, the carina a T6 being entire and triangular, the pale somewhat expanded front basitarsus with a row of elongate black densely plumose hairs beneath the white fringe of hairs (Figure 5a), the shallowly emarginate clypeal margin with a small, subapical median tubercle (Figure 6a), the body with pubescence entirely pale, the dense white tomentum of T3-T5 (Figure 4c), and S5 with postgradular area narrowly and deeply incised medially, almost separated into two halves (Figure 7c). It is most similar to other *Megachiloides* s. str. (*Megachile amica*, *Megachile oenotherae*, and *Megachile umatillensis*; i.e. the *oenotherae* species group) which all lack the pronounced median tubercle on the clypeal margin (Figure 6b). Male *Megachile amica* are typically smaller (10–12 mm), and have the carina of T6 less produced, with much dark pubescence on the apical terga, and S5 with postgradular area widely separated medially in basal half; males of *Megachile oenotherae* and *Megachile umatillensis* lack the black plumose hairs on the front basitarsis (Figure 5b), *Megachile oenotherae* has darker front tarsi, and much dark pubescence on the mesoscutum and mesoscutellum.

In addition to also possessing a relatively long tongue with the second labial palpomere at least 1.7× the length of the first (Figure 3e), the female of *Megachile chomskyi* can be distinguished by the 3-dentate mandible, with a small vestige of a tooth just posterior to the middle one, thus approaching a 4-tooth condition (Figure 8c), the relatively large size (13.5 mm), and the uniformly short dark hairs on T2-T4. It is also most similar to *Megachile amica*, *Megachile oenotherae*, and *Megachile umatillensis*. Females of *Megachile amica* are typically smaller (9-10 mm), and have dark hairs of varying length on T2-T4; females of *Megachile oenotherae* and *Megachile umatillensis* have an even longer tongue, the second labial palpomere at least 3× the length of the first (Figures 3f and g).

##### Description.

*Male*: Body length 13 mm, forewing length 10 mm. Head width 4.3 mm; head length 3.9 mm (Figure 4b). Tongue length 7.2 mm, first (i.e. basal) labial palpomere 0.55× length of second (Figure 3e). Intertegular distance 3.7 mm; distance between outer margins of tegulae 4.8 mm.

**Structure.** Compound eyes subparallel to slightly convergent below (Figure 4b). Lateral ocelli slightly nearer to compound eye than to edge of vertex (5:6). Mandibles 3-dentate, lower process of mandible slender, acute, subbasal in position. Clypeal margin narrowly shiny and impunctate, broadly and shallowly emarginate with a distinct median tubercle (Figure 6a). Gena as wide as compound eye in profile. F1 as long as broad, subequal in length to pedicel, slightly shorter than F2, F2 quadrate to very slightly longer than broad, F3-F8 longer than broad (2.5:2), apical segments more so (3:2), apical flagellomere more elongate, about twice as long as broad. Front coxal spine short, distinct, longer than broad, subacute with short dense patch of pale pubescence at tip, ventral surface otherwise bare, with small patch of reddish-brown subappressed bristles at base. T2 with shallow but distinct basal groove apical to gradulus, graduli of T3-T4 more distinct with carinate rims, basal grooves of T5 and T6 very deep, graduli with hyaline carinate rims. Apical margins of T2 and T3 slightly depressed, T4 and T5 very much so. T6 with carina entire, evenly triangular in dorsal view (Figure 4c), apical 1/3 slightly curved downward, median carinate teeth of apical margin very large and broadly rounded, closer to lateral teeth than to each other (2:3). T7 visible, triangular, pointed tip about as broad as long, gradulus deeply emarginated medially, triangular. S4 with apical margin very slightly emarginate medially with wide (1 OD) hyaline apical rim. S5 with pregradular area very thin medially, postgradular area narrow, deeply incised medially, almost separated into two halves, apical rim laterally produced with median hair tuft (Figure 7c). S6 with apical lobe emarginate medially, with lateral edges broadly angulate (~90°), postgradular area heavily sclerotized, setal patches rather widely divided, base of pregradular area slightly re-curved ventrally (several mites were found in the resulting cavity) (Figure 7b). S8 with lateral edges concave, apex rounded (Figure 7a). Gonocoxite sinuate in lateral view, with distinct ventral angle in basal third, narrowed basal to gonostylus region, ventral apical angle produced into small angular projection, dorsal apical surface covered with elongate hairs; penis valve relatively straight, slightly curved at tip and exceeding gonocoxite in length (Figures 7d and 7e).

**Colour and pubescence.** Integument black, tegula and apical tarsomere dark brown, front femur yellowish-brown ventrally, basitarsus yellow on outer surface, becoming light brown along edges, reddish brown ventrally, tibial spurs yellowish-brown, T6 with median apical teeth reddish brown, apical edge of S4 broadly (i.e. 1.5 OD) hyaline. Wings clear, faintly hyaline apically beyond veins, veins dark brown. Pubescence entirely pale yellowish-white on body, becoming somewhat paler to white on mesosoma ventrally, dense and entirely pale yellow on face below level of median ocellus, mesoscutum rather sparsely but uniformly pubescent, becoming slightly longer and denser at periphery and on scutellum, terga with mostly pale yellowish-white pubescence, long and rather dense on T1, sparser but long and suberect on T2, erect hairs becoming sparser on T3-T5, basal groove of T3 and T4 with narrow band of white tomentum, more extensive on T5 (Figure 4c), T6 thinly pale pubescent above carina, with elongate plumose hairs laterally and below carina, T2-T5 with dense white apical fascia; front basitarsus with row of elongate black densely plumose hairs beneath white fringe of hairs (Figure 5a).

**Surface sculpture.** Punctures fine and close on face, somewhat shallow but distinct on gena, becoming deeper but rather fine and close (<1pd) on vertex medially, more irregular sized but still close on vertex laterally, fine and very close on clypeus and on supraclypeal area, apical edge of clypeus narrowly shiny and impunctate, mesoscutum and mesoscutellum with punctures shallow but distinct, rather fine and uniformly close, surface dull, punctures becoming deeper on pleura below, tegula finely and closely punctate throughout, propodeum with shallow, fine punctures with i=0.5-1pd, triangle dull, smooth and impunctate, punctures fine over most of dorsal surface of metasoma, minute and very close on T2, larger but still close (i<1pd) on T3, larger on T4 with shiny i=1pd, larger and somewhat elongate on apical 1/3 of T5, fine and densely crowded on T6, coarse and close (i=1pd) on S1-S2, becoming sparser (i=3-4pd) in apical half of S3-S4.

*Female*: Body length 13–13.5 mm, forewing length 8.5 mm. Head width 4.0 mm; head length 3.3 mm (Figure 8b). Tongue length 7.2 mm, first (i.e. basal) labial palp 0.55× length of second (Figure 3e). Intertegular distance 3.3 mm; distance between outer margins of tegulae 4.2 mm.

**Structure.** Compound eyes very slightly convergent below (Figure 8b). Lateral ocelli as near to compound eye as to edge of vertex. Mandible 3-dentate, two apical teeth approximate, with small angle interrupting long cutting edge between 2^nd^ and inner teeth (Figure 8c). Clypeal margin smooth, very slightly produced in median third. Gena as wide as compound eye in profile. F1 as long as broad, and subequal in length to pedicel, longer than F2, F2 broader than long (1.5: 1.2), F3-F9 quadrate to slightly longer than broad, apical flagellomere more elongate (2.5:1.5). T2-T5 with shallow but distinct grooves across base, graduli carinate, apical margins slightly depressed, laterally only on T2. T6 very slightly concave in profile.

**Colour and pubescence.** Integument black, flagellum and tegula dark brown, tibial spurs yellow-brown. Wings clear, faintly hyaline apically beyond veins, veins dark brown. Pubescence mostly white on body, a few short dark hairs on vertex laterally, in apical half of T2 (basal to fasciae), more extensive on T3, occupying almost entire surface of T4 and T5 (Figure 9a), T6 with short, thick, black pubescence interspersed with thin, pale, longer hairs. S2-S5 with scopa white, black on S6, pale hairs sparse on clypeus and supraclypeal area, becoming denser and longer on face around bases of antennae, mesoscutum sparsely pubescent, with short erect hairs, more dense at periphery, more elongate on mesoscutellum and pleura.

**Surface sculpture.** Punctures fine and close on face, rather shallow on gena with surface shiny, deeper but rather fine and very close on vertex medially, slightly larger but still close on vertex laterally, quite coarse on clypeus in apical half, with i=≤1pd and reaching apical edge, closer laterally, much finer and closer in basal 1/3 and on supraclypeal area; mesoscutum and mesoscutellum with punctures shallow but distinct, rather fine and uniformly close, surface dull, punctures becoming deeper and slightly more separated on pleura below, tegula finely and closely punctate throughout, propodeum with shallow, fine punctures with i=0.5-1pd, triangle dull, smooth and impunctate; punctures fine over most of metasoma, minute and very close on T1 (dorsal surface) and T2, similar apically on T3 and T4, though interspaces larger in basal half (i=1-2pd), more coarse but still close (i=≤1pd) on T5, coarse and densely crowded to finely subrugose on T6, coarse and close on basal sterna, becoming slightly more separated on apically sterna, quite sparse on S6 (i=2pd).

##### Etymology.

It is my pleasure to name this species after Professor Noam Chomsky, Department of Linguistics & Philosophy at the Massachusetts Institute of Technology (MIT) for his many academic achievements and contributions as a linguist, philosopher, cognitive scientist, historian, political critic, activist and global champion of human rights and freedoms.

##### Distribution.

*Megachile chomskyi* is known from the state of Texas, United States.

## Discussion

*Megachile chomskyi* is morphologically very similar to *Megachile amica*, but differs from that species not only in a series of structural characters, but also in COI similarity by 7.8%; intraspecific variation for each species averaging less than 0.8% (n=3) (unpublished). The subgenus *Megachiloides* still remains one of the most problematic *Megachile* groups in North America, due in part to the large proportion of species described from one sex (Sheffield and Westby 2007), outdated keys and descriptions which are poorly illustrated. Sheffield et al. (2011) associated some of the sexes, and with the assistance of DNA barcoding, discovered that several of the species occur as melanistic forms previous recognized as valid species known only from the female. The relationship of *Megachiloides* with other subgenera is also still not resolved (Gonzalez 2008), though a phylogeny and full revision of the subgenus are forthcoming.

As is suspected for other members of the *oenotherae* species group of *Megachiloides*, *Megachile chomskyi* may be a floral specialist of Onagraceae (Evening-Primrose Family). Most of the specimens examined were collected on *Calylophus hartweggii* (Benth.) P.H. Raven., which also occurs in Arizona, Colorado, Kansas, New Mexico, and Oklahoma, and *Oenothera drummondii* Hook., which occurs in Texas, Florida, Louisiana, North and South Carolina. Therefore, it is possible that some specimens presently identified as *Megachile amica*, which ranges from Arizona, north to Kansas and southeast to Texas, are actually this species. In Texas, *Megachile chomskyi* has been collected from late April to June.

### Key to the *oenotherae* species group of *Megachiloides*

**Females**

**Table:** 

1	Tongue relatively short, extended length of glossum not reaching beyond mid length of metasoma (Figure 8a), second labial palpomere less than twice the length of the basal palpomere (Figures 3d and 3e)	2
–	Tongue long, extended length reaching almost to tip of metosoma (Figure 1), second labial palpomere at least three times the length of basal palpomere (Figures 3f and 3g)	3
2	Larger (13.5 mm), dark pubescence of T2 and T3 uniformly short and dense, maximum length less than half of width of pale apical fascia (Figure 9a)	*Megachile chomskyi* sp. n.
–	Smaller (≤11 mm), dark pubescence of T2 and T3 of intermixed length, length of longer, coarser dark hairs approaching width of apical fascia (Figure 9b)	*Megachile amica* Cresson
3	Larger (≥13 mm); pubescence on mesonotum entirely pale	*Megachile umatillensis* (Mitchell)
–	Smaller (11 mm); pubescence on mesoscutum and mesoscutellum with much black pubescence	*Megachile oenotherae* (Mitchell)

**Males**

**Table:** 

1	Fringe of front basitarsus with distinct ventral row of elongate, black densely plumose hairs (Figure 5a)	2
–	Fringe of front basitarsus entirely white, lacking black hairs (Figure 5b)	3
2	Larger (13 mm); clypeal margin shallowly emarginate, with small median tubercle (Figure 6a); carina of tergum 6 more produced, narrowly triangular; apical terga with pubescence almost entirely pale, tergum 5 with rather dense pale tomentum in basal half (Figure 4c)	*Megachile chomskyi* sp. n.
–	Smaller (10–12 mm); clypeal margin straight, lacking median tubercle (Figure 6b); carina of tergum 6 broadly rounded; apical terga with much black pubescence, tergum 5 lacking pale tomentum	*Megachile amica* Cresson
3	Larger (>13 mm); front basitarsus pale	*Megachile umatillensis* (Mitchell)
–	Smaller (9–10 mm); front basitarsus mostly dark	*Megachile oenotherae* (Mitchell)

## Supplementary Material

XML Treatment for Megachile (Megachiloides) chomskyi
